# Editorial: Narrow and general intelligence: embodied, self-referential social cognition and novelty production in humans, AI and robots

**DOI:** 10.3389/frobt.2025.1766766

**Published:** 2026-01-09

**Authors:** Sheri Markose, Tony Prescott, Georg Northoff, Emily Cross, Karl Friston

**Affiliations:** 1 University of Essex, Colchester, United Kingdom; 2 The University of Sheffield, Sheffield, United Kingdom; 3 University of Ottawa, Ottawa, Canada; 4 Eidgenossische Technische Hochschule Zurich, Zürich, Switzerland; 5 University College London, London, United Kingdom

**Keywords:** adaptive immune system, algorithmic takeover of biology, allostasis, artificial general intelligence (AGI), autonomy of goal setting, blockchain information processing, embodied self-referential genomic intelligence

A team of multi-disciplinary editors, whose views are reflected in the themes underscored in this Research Topic, has come together to help take stock of the phenomenal success of narrow Statistical Artificial Intelligence (SAI) and to examine new perspectives on achieving Artificial General Intelligence (AGI). The tenets of SAI, which remain contingent on the domain of knowledge uploaded in digital form, is often contrasted with a long-held view that AGI must emulate the human brain, which marks an apogee as a prototype for general intelligence (Prescott, 2024). Many of the editors are of the view that the general scope of human intelligence arises from the necessity of maintaining the homeostasis of life itself (Friston, 2010; Friston, 2013; Prescott and Jimenez-Rodriguez, 2025), under ever-changing and often hostile circumstances. Further, they hold the view that complex life manifests embodied self-referential information processing (Northoff et al., 2006) with empathic mirror systems for prolific self-other interaction, and with selfhood and autonomy of goal setting intrinsically configured and committed to a hack-free agenda to mitigate what is inimical to life.

Against this background, we will briefly review the dozen papers published in this Research Topic involving a wide array of perspectives of 37 authors. These papers have to date garnered over 50,000 views and downloads.

It is useful to start with the review paper by Wu et al. where the scene is set for AI generations that have unfolded over the last 7 decades from AI 1.0 to AI 4.0. These developments have been driven by a triad of factors relating to algorithms and software; chip technology and computing power; access and storage of voluminous data in static and real time mode. AI 1.0 is ground zero, with algorithms aimed to fully direct outcomes mostly based on logic and rules based inference. This phase was accompanied by internet technologies such as search engines, digital automation and data processing. The authors categorize AI 2.0 as encompassing Agentic AI, real-time online bots, and the advent of high-performance Graphics Processing Units (GPUs) and vast labeled datasets. This gave rise to deep learning and reinforcement learning, with convolutional and recurrent neural networks achieving breakthroughs in vision, language, and control. The third AI 3.0 generation marks the embedding of digital intelligence in an embodied physical agency of robots, where AI operates in material spaces as in autonomous vehicles and other utilitarian cobots.

The fourth generation AI 4.0 is set to coincide with aspirations of AGI with controversial notions of machine sentience, with self-capable, adaptive selection of goals and the wherewithal to evolve programs to achieve goals autonomously. Markose. points out that the lack of AI alignment (Bostrom, 2014; Russell, 2019) with human values and goals encountered in agents capable of setting their own goals is not unique to AI, but is a problem that lies at the foundations of civil society. Conflicting or adversarial goals of agents and their accompanying actions that are inimical to life must be kept in check for the survival of the human condition. On the other hand, providing AIs that are value-aligned with some awareness, and capacity for moral reasoning, could make them safer and better able to recognise and mitigate risks (Wallach, 2008).

The Markose. perspective on genomic intelligence—underpinned by the algorithmic takeover of biology within a uniquely encoded system—is that there are lessons to be learnt on the alignment problem from the evolution of general intelligence in complex life. The view here is that alignment to life and the design of selfhood has been solved in formal ways that can be explicated using Gödel logic, and with recent developments in cryptography with the blockchain. The principles involved here can be conjectured to maintain the immutability of original protein coding blocks against internal and external bio-digital adversaries within an evolvable and unbroken chain of life.

It has become popular to refer to self-improving code-based systems as Gödel machines in AGI frameworks, which are necessarily end-to-end self-assembly programs as in life (Schmidhuber, 2006; Zhang et al., 2025). Markose. suggests that this misses the point of Gödel logic, which is embedded in complex life first found in the adaptive immune system, AIS, of jawed fish 500 mya and latterly in the mirror neuron systems of primates. About 85% of expressed genes that can be identified as online self-assembly machines that create the morphology and phenotype of a multicellular organism can be viewed as its theorems. These are mapped offline in AIS ‘Thymic Self’ à la Self-Representation (Self-Rep) structures from the Recursive Function Theory of Gödel-Turing-Post. The purpose of this is to recursively identify non-self codes, especially of digital adversaries wielding the negation operator, which are potentially uncountable infinity. A corresponding open-ended capacity to detect changes to self-codes - known to be found only in the AIS and the human brain in a process of prolific predictive coding—is empowered by the Recombination Activation Gene operators. In a bold hypothesis, Markose (2022), Markose (2021) states that the Gödel Sentence is known to have little relevance in the real world, but is ubiquitous in complex life as a hashing algorithm (to adopt the language of blockchains) that enables embodied self-referential intelligence to detect any misalignment or negation of life’s self-codes. This is accompanied by an arms race in novelty or surprises in a game with the viral/digital adversary, first identified by the game theorist Binmore (1987) in the archetype of Godel’s Liar, to maintain the primacy of life codes. This self-regulation is achieved internally or by human external phenotypical interventions with human artifacts often in a structure of a perpetual Gödelian arms race.

This nicely takes us to other papers that investigate self-regulation and embodied intelligence within humans and AI systems. The research paper of Verchure et al. investigates the self-regulatory processes not through code-based smart controls, which can suffer misalignment by attacks by internal or external bio-malware as per Markose., but via the notion of allostasis, modelled by dynamical equations, whereby multiple physiological parameters are monitored and controlled “to maintain the stability of the integrated self rather than its parts”. In particular, they consider how the mammalian brain conducts allostatic regulation of action, as an extension of the principle of homeostasis, using a predictive and adaptive multi-layered control architecture (see also Prescott and Jimenez-Rodriguez, 2025). They deploy an allocentric synthetic agent in a virtual environment and test the dynamical properties of the neural mass allostatic model with internal needs such as heat and hydration to be fulfilled in three scenarios. These relate to (1) open field rodent behavior, (2) where adaptation in navigation is needed, and (3) when criticality reset optimizes the interoceptive-driven decision-making process. They find, that though environmental stressors challenge the capacity to fulfil the agent’s internal needs, the neural mass model with its self-regulatory dynamics achieves a robust balance in this regard.

The perspective paper by Caucke et al. explores how our understanding of the prolific capacity of social cognition in humans can help build the same capacities in robots. They review well-known theories on embodied self with self-knowledge - both from the interoceptive internal environment and the external environment, via the sensory motor cortex that undergirds physical situatedness. The use of self-knowledge as the basis of social cognition, empathy and action prediction of other similarly wired-up conspecifics and the strategic necessity of the Sally-Ann problem of false beliefs relating to perception of negation - are discussed. The authors are keen to emphasize that as human social cognition depends on some degree of individual autonomy, remote or externally controlled robots do not engage in social cognition. Likewise, they state that swarm robots that can self-organize along a well-defined and externally limited action set do not have autonomy in the choice of goals or actions. They touch on the fundamental problem of coordination and cooperation when robot behaviors are mutually predictable by robots themselves via good internal models of the other. This requires that the robots do not engage in unpredictable actions that are adversarial or disruptive of what is mutually predictable. While specific robots can have their autonomy limited in order to be cooperative, as indicated in the seminal work of Binmore (1987), digital adversaries cannot be eliminated in general and robots like humans must be capable of detecting Liars/adversaries and enter into arms races with them to preserve autonomy of self.

In Ryan. perspective paper, the embodied and ecological approach to intelligence, with the former based on the framework of the Learning Intelligent Decision Agent (LIDA), is used to understand novelty and improvisation in music. For this Ryan draws on the Jeff Pressing model which entails the knowledge base comprised of cognitive units of objects and processes stored in long term memory of the musician. Processes bring about changes in features and objects and all of these aspects interact with one another in complex ways. Ryan favors the LIDA approach rather than AGI as LIDA’s framework of cognitive cycles of learning, perception, and action engages high and low levels of cognition of an autonomous agent without a specific problem-solving goal typically associated with AGI. In future works Ryan aims to show a LIDA/Pressing robot for music improvisation compared with existing improvising AI machines.

Pontes-Filho et al. challenge the view that AGI should emulate human-level intelligence and instead argue that the starting point of AGI should be at a much lower level, which they call Neuroevolutionary AGI (NAGI) when learning occurs through sensory experience. They propose at a minimum - a body and a reactive environment - where evolutionary complexification can happen. From a randomly initialized spiking neural network, they posit that learning occurs with adaptive synapses which control binary signals (excitatory or inhibitory) that propagate through reconfigurable network topology. Hence, this method has been called Neuroevolution of augmented topologies (NEAT). This method, though comparable, does not follow gradient descent of deep neural networks for reinforcement learning tasks. NAGI is successfully tested on three tasks: food foraging, emulation of logic gates, and cart-pole balancing. This approach, while promising, begs the question of how an AI learning to do preassigned tasks can achieve AGI ambitions, which typically include autonomous choice of goals themselves.

Johansson. aims to advance AGI by developing what he calls Machine Psychology by harnessing operant learning psychology based on behavioral changes due to the consequences of actions, integrating it with the Non-Axiomatic Reasoning System (NARS). NARS has been built with sensorimotor reasoning at its core, enabling it to process sensory data in real-time and respond with appropriate motor actions. NARs is equipped to be an efficacious inference system with limited knowledge and resources, a condition that is often true for real-world scenarios. Combining the two is an apt example of learning by doing, though Johansson brings in the Skinnerian behavioral triad of stimulus, response, and reward and an additional *establishing operation* (EO), which can enhance or mitigate the stimulus and make the response more (less) likely. Well-known critiques of Skinner—such as that by Chomsky—exist on how language acquisition, for instance, requires more than operant conditioning. The tasks used to test out Machine Psychology, though successful, fall far short of the prowess expected by AGI. The clear advantage of NARS is that it can eschew large data sets, unlike traditional AI systems, as NARS operates effectively under conditions of insufficient knowledge and resources.

In robot intelligence Chen et al. like Johansson. propose the use of fast and frugal heuristic decision making, as in humans, conjectured to lead to more robust inference in real-time systems in which rapid decision making is essential. Bounded rationality solutions of Herbert Simon that rely on satisficing rather than optimization underpins the branch of learning called active perception in robots, which uses less data than onerous deep learning solutions. The authors use human decision makers to solve simulated treasure hunt problems in a virtual environment to derive efficacious decision rules as time and other pressures, such as impediments to visual perceptions (fog), are increased. The most efficacious human strategies discovered from human studies are then implemented on autonomous robots equipped with vision sensors. Results show robust performance of robots using the heuristic toolbox, when compared with known optimization algorithms that fail to complete the search for treasures under unanticipated adverse conditions.

In a second paper, Johansson. shows how Arbitrarily Applicable Relational Responding (AARR)—which has been considered to be a particularly human facility for flexible and contextual learning—can be captured by suitably designed AI systems. He aims to achieve this by combining Non-Axiomatic Reasoning System (NARS) used for learning under uncertainty with the behavioral psychology account of AARR, which enables NARS to derive symbolic relational knowledge directly from sensorimotor experiences. He shows how key properties of AARR (mutual entailment, combinatorial entailment, and transformation of stimulus functions) can emerge from NARS’s inference rules and memory structures. The claim is that this can pave the way to AGI, though there is some considerable work that needs to be done to bring this to fruition.

In the final three papers that are reviewed here, applications of extant AI for specific tasks are considered, or some new enhancements have been incorporated to achieve more efficacious performance.

Zhai et al. use a multi-modal and multi-level approach for enhanced human-robot interactions. Multi-modal intelligence is a desired AGI characteristic, as a combination of visual, auditory, and language-enabled human intelligence gives enhanced experience and performance, and impairment in any of these modalities places an individual at a considerable disadvantage in life. However, in a robot setting, the architecture of multimodal intelligence is considerably more complicated when combining, say, computer vision for object identification with natural language processing for named entity identification. This is especially the case in settings like social media postings, where images and texts are short and prone to noise, making it harder to achieve feature selection and identify relevant information. The authors develop a multimodal named entity recognition (MNER) architecture in which the neural network can extract useful visual information for enhancing semantic understanding and subsequently improve entity identification. Twitter data sets with pictures and text are used for experiments and to test out their MNER model. The enhanced performance of their MNER—when compared to other multi-modal models—comes at the price of slower operations.

Babushkin et al. investigate how handwriting, which is the outcome of multimodal inputs in humans, can be evaluated by an AI such as a temporal convolutional neural network (TCN). The use of AI is seen to overcome biases that humans have in their assessment of handwriting; there are educational, forensic, and technological contexts where AI can provide a more efficient and accurate service. For their AI experiment, the handwritten documents of an identical text—designed to include all possible orthographic combinations of Arabic characters—were done by 50 human subjects, and three experts were used to categorize the produced text into different legibility scores. The TCN is trained to classify the documents into different legibility categories. The results show that while the TCN model trained on stylus kinematics features demonstrates relatively high accuracy (around 76%), the addition of hand kinematics features significantly increases the model accuracy by approximately 10%.

In the final paper Binzagr and Abulfaraj. aim to improve traditional machine learning methods for diagnosing Alzheimer’s disease (AD) from MRI scans, claiming that CNN architectures have problems with detecting AD due to overfitting. In addition to the CNN, the authors incorporate two other components into their new framework. These include a generalized self-attention (GSA) score, which gives a global assessment of interdependence across spatial and channel dimensions while filtering out irrelevant details, and an extreme learning machine (ELM) classifier, employed to categorize AD. Note, the GSA blocks are placed on an InceptionV3 network, which is a directed acyclic graph (DAG) network that has 316 layers and 350 links that include 94 as convolutional layers. In-depth experiments on two benchmark datasets demonstrate that the proposed InGSA achieves superior performance compared to the state-of-the-art techniques.

This Research Topic provides a valuable snapshot of where thinking about AGI is in the early-mid 2020s. Some crosscutting themes in the research and review articles laid out above are agency, autonomy, and autopoiesis, read in their broadest terms. Indeed, ‘codeopoiesis’ or how code-based genomic intelligence achieves self-organization (Markose, 2022) as in blockchains is underscored to reflect new developments in autonomous AI.
